# Supplementary figures and images for: Structuring of Cold Pressed Oils: Evaluation of the Physicochemical Characteristics and Microstructure of White Beeswax Oleogels
Source: Gels. 2023 Mar 13;9(3):216. doi: 10.3390/gels9030216 (PMC10048366; doi:10.3390/gels9030216)

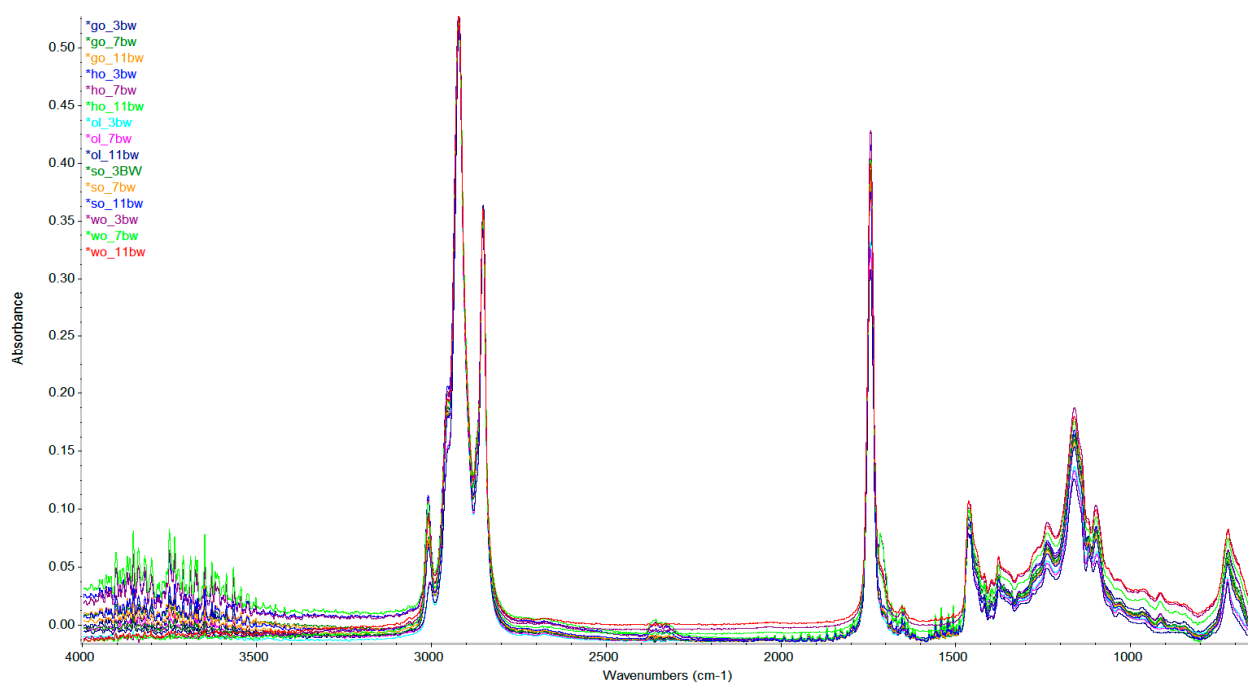

**Figure S1:** Graphic representation of spectra overlay

Supplement: Supplementary file 1 [file gels-09-00216-s001.zip › gels-2266409-supplementary.pdf]
